# Data Mining in Translational Bioinformatics

**DOI:** 10.1155/2014/656519

**Published:** 2014-06-12

**Authors:** Xing-Ming Zhao, Jean X. Gao, Jose C. Nacher

**Affiliations:** ^1^School of Electronics and Information Engineering, Tongji University, Shanghai 201804, China; ^2^Department of Computer Science & Engineering, University of Texas, Arlington, TX 76019, USA; ^3^Department of Information Science, Faculty of Science, Toho University, Chiba 274-8510, Japan

Translational bioinformatics is an emerging field that aims to exploit various kinds of biological data for useful knowledge to be translated into clinical practice. However, the flooding of the huge amount of omics data makes it a big challenge to analyze and to interpret these data. Therefore, it is highly demanded to develop new efficient computational methodologies, especially data mining approaches, for translational bioinformatics. Under these circumstances, this special issue aims to present the recent progress on data mining techniques that have been developed for handling the huge amount of biological data arising in translational bioinformatics field.

In data mining, one of the most important problems is how to represent the data so that the computational approaches could handle these data appropriately. In this special issue, B. Gan et al. utilized the latent low-rank representation to extract useful signals from noisy gene expression data and then classified tumors with sparse representation classifier and obtained promising results on benchmark datasets. C. Zhao et al. proposed a new feature representation of facial complexion for diagnosis in traditional Chinese medicine and achieved high recognition accuracy. G. Zhang et al. formulated the skin biopsy image annotation as a multi-instance multilabel (MLML) problem and automatically annotated the skin biopsy images with a sparse Bayesian MLML algorithm based on region structures and texture features. Except for feature extraction, feature selection is also very important in data mining. Z. Ji et al. proposed a particle swarm optimization-based feature selection approach to predict syndromes for hepatocellular carcinoma and improved diagnosis accuracy. With the accumulation of various data in translational bioinformatics, it is becoming a challenging task for traditional intelligent approaches to handle and interpret these data; S. Li et al. presented a survey on the recent progress about the hybrid intelligences and their applications in bioinformatics, where the hybrid intelligence is more powerful and robust compared with traditional intelligent approaches.

The rapid accumulation of various kinds of biological data requires more powerful statistical approaches to extract useful signals from the huge amount of noisy data. L. Sun et al. built a new pipeline to investigate the DNA methylation profiles in male and female nonagenarians/centenarians and identified some differentially methylated probes between male and female nonagenarians/centenarians, which provide insights into the mechanism of longevity gender gap of human beings. Z. Teng et al. developed a new algorithm to predict protein function based on weighted mapping of domains and GO terms, which outperforms other popular approaches on benchmark datasets. J.-L. Huang et al. presented an online cross-species comparative system to identify conserved and exclusive simple sequence repeats within model species, which can facilitate both evolutionary studies and understanding of gene functions. L. Guo et al. proposed a new approach to identify microRNAs (miRNAs) associated with breast cancer and found that miRNA gene clusters demonstrate consistent deregulation patterns despite their different expression levels, which may provide insights into the regulatory roles of miRNAs in tumors.

Recently, network biology is becoming a promising research field by organizing different kinds of data into a network representation. T. Jacquemin et al. proposed a new approach to identify disease associated protein complexes based on a heterogeneous network that consists of a disease similarity network and a tissue-specific protein-protein interactions network and successfully found disease associated complexes. X. Li et al. proposed a new pipeline to detect symptom-gene associations by integrating multiple data sources and found some potential disease genes. It is known that DNA mutations will affect gene expression. However, it is difficult to know which mutations will affect the gene expression and how the genes are regulated within the biological system. D. Kim et al. developed a novel approach that can both identify the Quantitative Trait Loci and infer the gene regulation network and successfully identified the genes associated with psychiatric disorder. R. Zhang et al. presented a new approach to identify the pathways linking TGF *β* to ovarian carcinoma immunoreactive antigen-like protein 2 (OCIAD2) by exploring the pathway bridge, and the resultant pathway explained how TGF *β* affects the expression of OCIAD2 in cancer microenvironment.

*Xing-Ming Zhao* *Jean X. Gao* *Jose C. Nacher*

